# The efficacy of parecoxib in improving pain after total knee or total hip arthroplasty: Systematic review and meta-analysis

**DOI:** 10.1097/MD.0000000000030748

**Published:** 2022-09-23

**Authors:** Chuan Hong, Hai-Yan Xie, Wu-Kun Ge, Min Yu, Shuai-nan Lin, Cheng-Jiang Liu

**Affiliations:** a Department of Orthopedics, Ninghai First Hospital, Ningbo, Zhejiang, China; b Department of Pharmacy, The Third Hospital of Quzhou, Zhejiang, China; c Department of Pharmacy, Ninghai First Hospital, Ningbo, Zhejiang, China; d Department of General Medicine, Affiliated Anqing First People’s Hospital of Anhui Medical University, Anhui, China.

**Keywords:** analgesia, hip arthroplasty, knee arthroplasty, parecib

## Abstract

The cyclooxygenase-2 (COX-2) selective inhibitor parecoxib is widely used in the treatment of pain and inflammation. Parecoxib has been adopted for use for postoperative analgesia following a range of surgical procedures (orthopedic, general, gynecological, and dental surgery). Total knee or total hip arthroplasty (THA) surgery is mostly done in older patients, so postoperative analgesics need to be used more carefully, and the safety and efficacy of parecoxib in this type of surgery need to be further verified. The aim of this study was to investigate the effects of parecoxib on patient safety, cumulative morphine consumption and was at 24 and 48 hours in the analgesic treatment of total knee or THA for meta-analysis and systematic review, with few studies in this area so far.

**Methods:**

We searched the Online Database Cochrane Library, PubMed, Web of Science, EMBASE, and CBM (SinoMed), CNKI, VIP, WANFANG up to January 2021. According to the value of *I*^2^, the random-effect model or fixed-effect model was supposed to combine data from studies, respectively. Publication bias was assessed through funneling plot and Beggs test. Review Manager 5.3 and Stata 16.0 software were applied to perform the statistical analyses.

**Results:**

Eleven RCTs which involved 1690 participants were included in this study. The meta-analysis indicated parecoxib sodium could not significantly reduce the incidence of adverse events after total knee or THA compared with placebo. There was no statistical significance in incidence of nausea and vomiting. 24 hours resting VAS score was statistically significant between the group. The 48-hour resting VAS scores did not indicate a significant difference between the groups.

**Conclusion:**

Parecoxib can reduce the incidence of adverse events after total knee or total hip surgery to some extent but cannot reduce the incidence of nausea and vomiting. Twenty-four hour postoperative analgesia is better than placebo, but 48 hours after operation analgesia is the same as placebo.

## 1. Introduction

It is estimated that 310 million patients worldwide undergo surgery every year.^[1]^ After >50 years of clinical practices, the therapeutic effect of artificial joint replacement has been fully affirmed and has developed into a reliable treatment. The 25-year combined survival rate of total knee or total hip replacement is 77.6%.^[2]^ In the United States, 1 million total knees or total hip arthroplasty (THA) and total knee arthroplasty (TKA) are performed each year,^[3,4]^ which is expected to increase in the next few years. The incidence of complications in total knee or THA is 3.2% to 8.0%.^[5]^ How to reduce the safety and pain after total knee or THA is an effective way to improve postoperative complications.

Morphine is an opiate receptor agonist, which has a good effect on all kinds of pain. The most common adverse reaction is nausea and vomiting, and extensive use can also make patients addicted.^[6]^ Use of opioids after orthopedic surgery varies greatly, and there is no consensus on the establishment of appropriate nursing standards.^[7]^ Many patients who take opiates before operation continue to use opioids after joint replacement. Some patients who have not used opioids still use opioids, but the continuous use of opioids has nothing to do with joint pain.^[8]^ There is no consensus on the best method of anesthesia and analgesia for total knee or THA.^[9–14]^ The purpose of our clinical practice is to control postoperative pain effectively and minimize the risk of using opioids.^[15]^

Pareoxib is the prodrug of valdicoxib, and valdicoxib is a selective cyclooxygenase (COX)-2 inhibitor in the clinical dose range, which has been widely used in postoperative analgesia.^[16,17]^ Studies have demonstrated that COX-2, as an isomer of cyclooxygenase, is induced by preinflammatory stimulation, so it is speculated that COX-2 plays the most important role in the synthesis of prostaglandin-like transmitters related to pain, inflammation and fever.^[18]^ Dozens of studies have shown that in the absence of pain, adverse events and other negative factors, early activity after joint replacement can shorten the hospital stay of about 1.8 days, and there are positive benefits to achieve early activity within 24 hours after operation.^[19]^ However, it is not clear whether pareoxib can effectively reduce the negative factors after knee or hip arthroplasty. This study is based on a randomized controlled meta-analysis and systematic review to evaluate the clinical role of pareoxib in pain relief of total knee or THA.

## 2. Materials and Methods

### 2.1. Inclusion and exclusion criteria randomized controlled trial

#### 2.1.1. Research object

For patients who receive THA or TKA, their race, nationality and course of disease are not limited.There are no ethical issues with our article.

#### 2.1.2. Intervention measures

The observation group was treated with pareoxib, and the control group was treated with placebo. Routine anesthesia was used in both groups, and the results were consistent between the 2 groups.

#### 2.1.3. Outcome index

① Overall adverse events, ② nausea and vomiting events, ③ 24-hour resting VAS score, and ④ 48-hour resting VAS score (see Table 1).

#### 2.1.4. Exclusion criteria

① Non-Chinese and English literature; ② lack of analytical data, which could not be obtained by contacting the original author; ③ repeated publication of literature.

### 2.2. Literature retrieval strategy

Pubmed, CochraneLibrary, Embase, China Medical Database, related system reviews, bibliography of clinical guidelines, and clinical trial registry were searched in the database. In addition, the reference parts of each study were also searched. The key words included pareoxib, hip arthroplasty, knee arthroplasty, and pain relief. The search was only restricted in English and Chinese publications, and we checked the reference lists of retrieved articles and relevant reviews for additional published and unpublished data.

### 2.3. Literature screening and data extraction

The 2 researchers independently screened the literature, extracted the data and cross-checked them, and if there were any differences, they would be resolved through discussion. When screening the literature, first read the title, after excluding the obviously irrelevant literature, further read the abstract and the full text to determine whether to include it or not. If necessary, contact the original research author by email or telephone to obtain undetermined information that is very important to this study. The contents of data extraction include: ① the basic information included in the study: research topics, first authors, published journals, etc; ② baseline characteristics and intervention measures of the subjects; ③ key elements of bias risk assessment; ④ outcome indicators and outcome measurement data concerned.

Two researchers independently evaluated the bias risk included in the study and cross-checked the results. Bias risk assessment uses the RCT bias risk assessment tool recommended by Cochrane manual 5.1.0.

The data were analyzed by RevMan5.3 software and Stata16. The mean difference was used as the effect analysis statistic for the measurement data, and the risk ratio (RR) was used as the effect analysis statistic for the 2-category variables, and each effect quantity provided its 95% CI. The heterogeneity among the included studies was analyzed by χ^2^ test (the test level was α = 0.1). Meanwhile, the heterogeneity was quantitatively judged by *I^2^*. If there is no statistical heterogeneity among the studies, the fixed-effect model is used for Meta-analysis; if there is statistical heterogeneity among the studies, the source of heterogeneity is further analyzed. After excluding the influence of obvious clinical heterogeneity, the data are analyzed by random-effect model for Meta-analysis. The level of Meta-analysis was set as α = 0.05. The obvious clinical heterogeneity was treated by subgroup analysis or sensitivity analysis, or only descriptive analysis.

## 3. Result

### 3.1. Research inclusion and exclusion process

**Figure 1.**
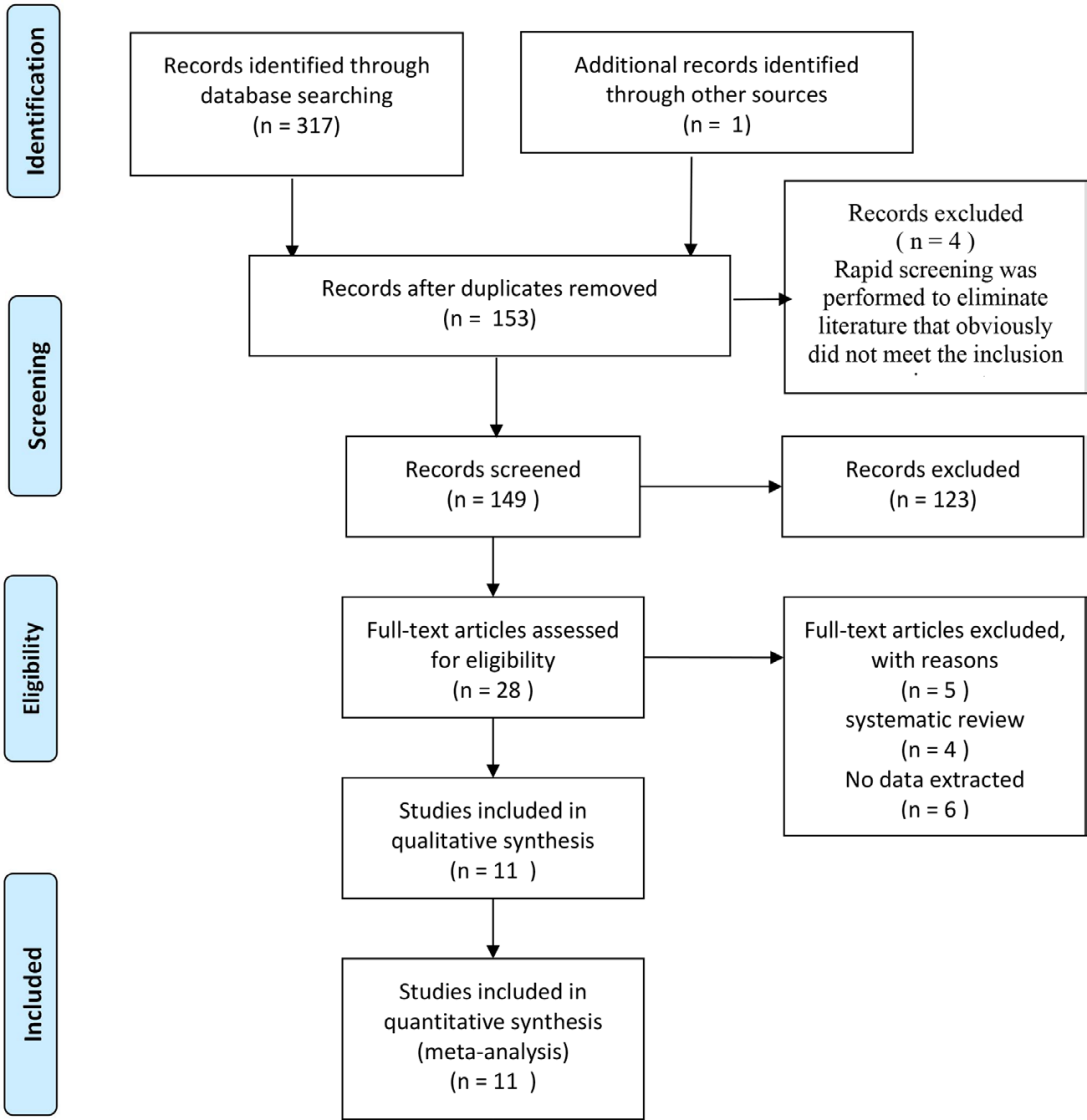
PRISMA flow diagram of the literature search process. PRISMA = Preferred Reporting Items for Systematic Reviews.

A total of 318 related articles were obtained in the initial examination. After layer-by-layer screening, 13 RCTs (including 1868 patients) were included. The literature screening process and results are shown in Figure 1.

### 3.2. The basic characteristics of the inclusion study and the results of bias risk assessment

The 3-item scale of Jadad was used to assess the quality of included studies (Table 1).^[33]^ This instrument is referred to as the “Jadad scale.” Scale scores can range from 0 to 5 points, with higher scores indicating better quality (Table 2).

**Table 1:** Basic characteristics of literature included.

Included studies	Age of patients	Sample size	Intervention		Outcomes measures
	(T/C)	(T/C)	T	C	
Ke et al^[20]^	53.79 ± 12.46/54.39 ± 11.93	69/72	40 mg parecoxib intravenously	normal saline at the same time	①②③④
Wichai et al^[21]^	68.05 ± 9.75/64 ± 7.41	40/40	40 mg parecoxib intravenously	Celecoxib is taken orally	①②
T Philip et al^[22]^	68 ± 13/64 ± 13	64/70	40 mg parecoxib intravenously	placebo	①②
Valéria et al^[23]^	62 ± 11/63 ± 11	22/22	40 mg parecoxib intravenously	placebo	①②
Hui et al^[24]^	55.19 ± 10.97/57.22 ± 12.51	48/46	40 mg parecoxib intravenously	normal saline at the same time	①②③④
Du et al^[25]^	51.5 ± 8.9/52.5 ± 10.6	30/30	40 mg parecoxib intravenously and triamadol oral	Cocktail injection of joint cavity and triamadol oral	①②③④
Zhuang et al^[26]^	68.52 ± 7.26/67.08 ± 7.69	123/123	40 mg parecoxib intravenously	placebo	①②
Essex et al^[27]^	66.2 ± 6.65/67.6 ± 4.96	58/58	40 mg parecoxib intravenously	placebo	①②
Dong et al^[28]^	69.6 ± 6.5/70.5 ± 6.9	310/310	40 mg parecoxib intravenously	normal saline at the same time	②
Du et al^[29]^	68.5 ± 7/68.9 ± 7.2	35/34	20 mg parecoxib intravenously Tramadol and celecoxib loral	Tramadol and celecoxib oral	①②
Dai et al^[30]^	63.2 ± 8.5/65.2 ± 7.9	43/43	40 mg parecoxib ropivacaine and Dizocine intravenously	ropivacaine and Dizocine intravenously	①②
Bian et al^[31]^	66.64 ± 7.27/66.12 ± 8.34	46/42	40 mg parecoxib intravenously	normal saline at the same time	①②③④
Sarridou et al^[32]^	70.31 ± 9.69/70.73 ± 18.27	45/45	40 mg parecoxib intravenously	placebo	③④

①overall adverse events ② nausea and vomiting ③ 24 hours resting VAS score ④ 48 hours resting VAS score.

**Table 2:** Quality assessment of included studies.

Study (year)	Randomization	Double blinding	Withdrawals/dropouts	Jadad Score
Ke 2019	Appropriate	Low risk	Yes	4
Wichai 2010	Not clear	Unclear risk	Yes	3
T Philip 2003	Not clear	Unclear risk	Yes	3
Valéria 2007	Appropriate	Low risk	Yes	4
Hui 2018	Appropriate	Low risk	Yes	4
Du 2014	Appropriate	Low risk	Yes	4
Zhuang 2020	Not clear	Unclear risk	Yes	3
Essex 2018	Not clear	Unclear risk	Yes	3
Dong 2017	Not clear	Unclear risk	Yes	3
Du 2011	Appropriate	Low risk	Yes	4
Dai 2017	Appropriate	Low risk	Yes	4
Bian 2018	Appropriate	Low risk	Yes	4
Sarridou 2015	Appropriate	Low risk	Yes	4

Scale scores can range from 0 to 5 points, with higher scores indicating better quality.

### 3.3. Meta-analysis result

#### 3.3.1. Overall adverse event rates

**Figure 2.**
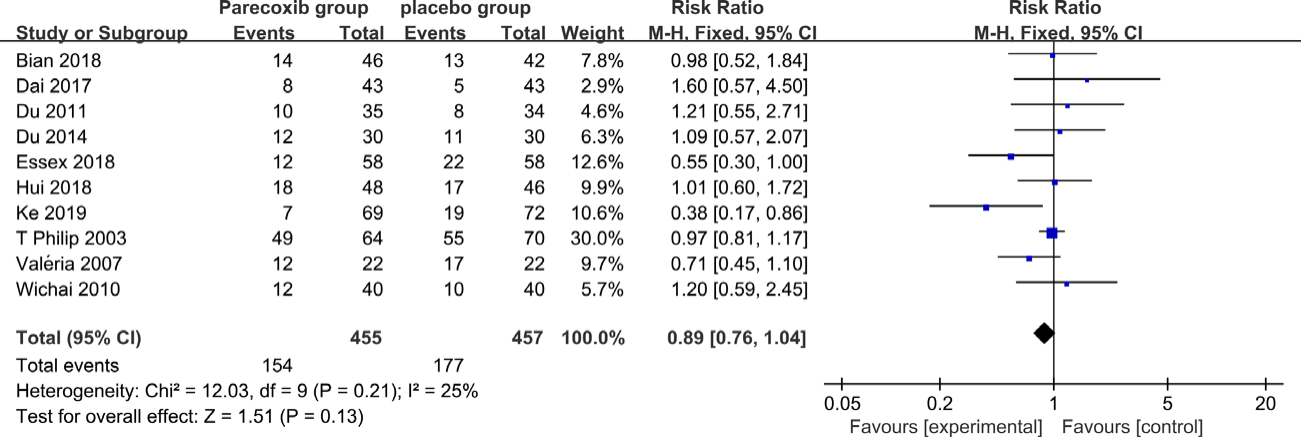
Forest plot of overall adverse event rates.

The 10 RCTs in this study have been tested for heterogeneity, and Q test *P* = .21 > .01, *I^2^* = 25%<50%, suggesting that there is mild heterogeneity among the selected literatures in this study, and the fixed effect is selected for the combined effect,finally RR = 0.89 (0.76–1.04, Fig. 2), indicating that the overall adverse event rate of parecoxib sodium after knee or hip joint surgery is only 0.89 times that of the placebo group, but Not statistically significant (Z = 1.59, *P* = .13 > .05), suggesting that although pareoxib sodium can reduce the incidence of adverse events after hip surgery, the degree of reduction is not statistically significant, that is, from a statistical point of view, there was no significant difference in adverse events between pareoxib sodium and placebo.

#### 3.3.2. Funnel chart

**Figure 3.**
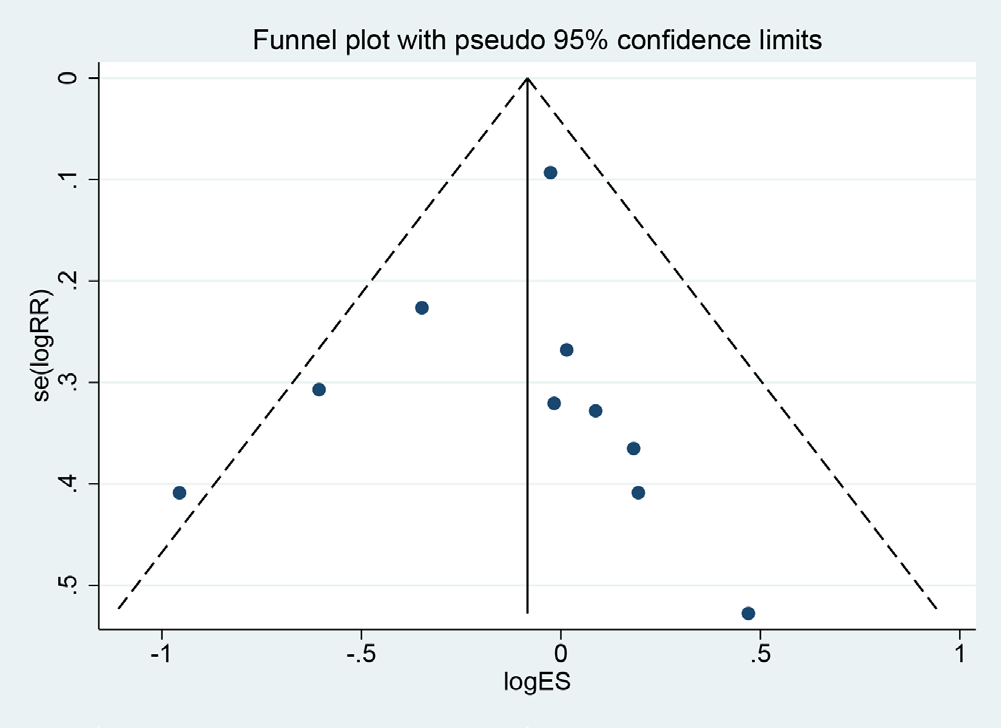
Funnel chart of overall adverse event incidence.

By drawing a funnel chart to investigate whether there is publication bias in the 10 RCTs of this study, it is concluded that the funnel chart is symmetric (*P* = .695 > .05 from Egger test) and no publication bias, which indicates that the conclusion of this study is accurate and reliable (Fig. 3).

#### 3.3.3. Incidence of nausea and vomiting events

**Figure 4.**
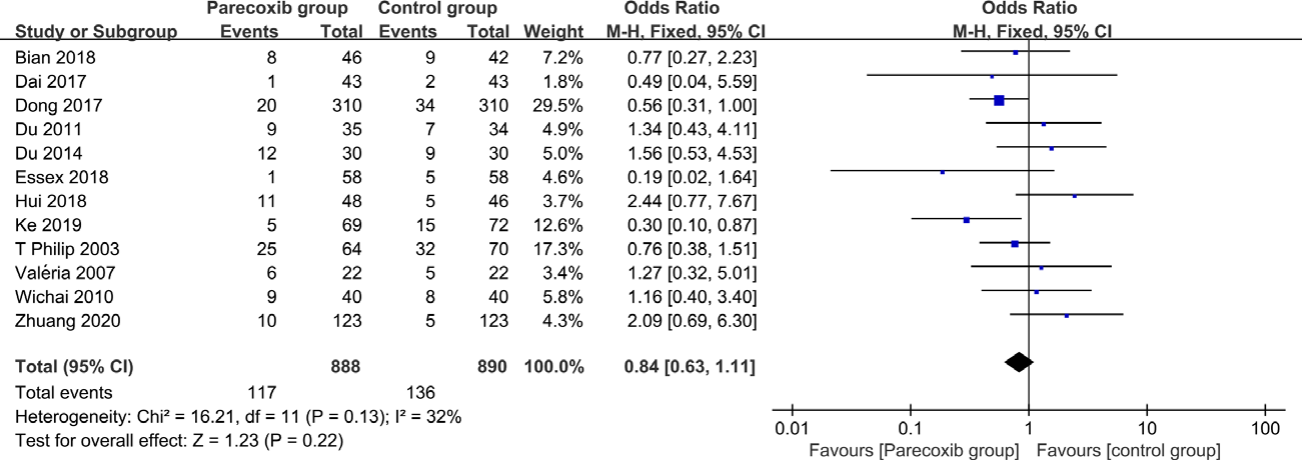
Forest plot of nausea and vomiting event rates.

The 12 RCTs in this study were tested for heterogeneity, *I^2^* = 32%<50%, and *P* = .13 for Q test was >0.01, suggesting that there is a slight heterogeneity among the documents selected in this study, and the fixed effect is selected Perform a combined effect size, finally RR = 0.84 (0.63–1.11, –4), which means that the overall adverse event rate of parecoxib sodium after knee or hip joint surgery is only 0.84 times that of the placebo group. It is statistically significant (Z = 1.23, *P* = .22 > .05), suggesting that although pareoxib sodium can reduce the incidence of adverse events after knee or hip surgery, the degree of reduction is not statistically significant, that is, from a statistical point of view, there was no significant difference in nausea and vomiting between pareoxib sodium and placebo (Fig. 4).

#### 3.3.4. Funnel chart

**Figure 5.**
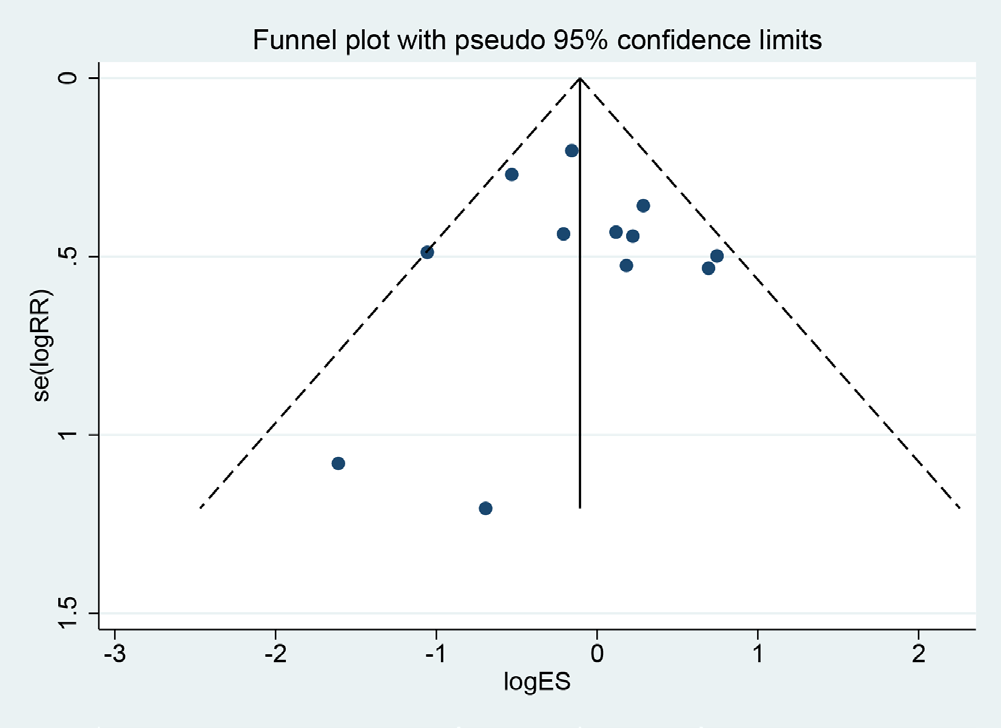
Funnel chart of nausea and vomiting.

By drawing a funnel chart to investigate whether there is publication bias in the 10 articles of this study, it is concluded that the funnel chart is symmetrical (*P* = .896 > .05 from Egger test), and there is no publication bias conclusion, suggesting that the conclusions of this study are accurate and reliable (Fig. 5).

#### 3.3.5. Consistency test of VAS baseline period

**Figure 6.**
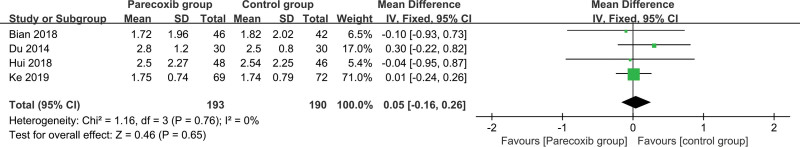
Baseline forest plot of preoperative VAS. VAS = Visual analogue scale.

Before performing meta-analysis, it is necessary to ensure that the baseline periods of the 2 groups of VAS are consistent, so that subsequent meta-analysis can be performed. Finally, 4 articles provided VAS baseline data, and the results are as follows. From the above forest diagram, we can clearly see that there is no heterogeneity in the VAS baseline period difference (effect size) between the 2 groups (*I^2^* = 0%<50% and Q test *P* = .76 > .1, Fig 6), and the fixed effects are combined with the baseline period. Finally, the combined effect size is (z = 0.46, *P* = .65 > .05), that is, in the baseline period, there is no difference in the VAS scores between the 2 groups, and subsequent Meta-analysis can be performed.

#### 3.3.6. 24-hour resting VAS score

**Figure 7.**
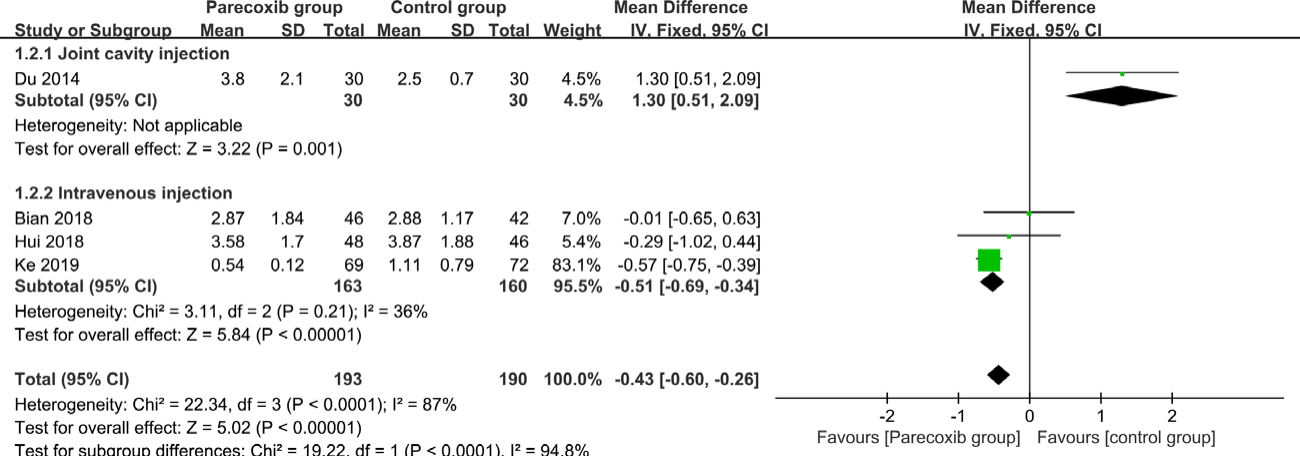
Forest plot of 24 hours resting VAS score. VAS = Visual analogue scale.

The control group was divided into 2 groups according to different administration methods, 1 group was intraarticular injection, and the other group was intravenous injection. The heterogeneity of the intravenous injection group (*I^2^* = 36%, *P* = .21 > .1) is not statistically significant, and the fixed-effect model is selected to combine the effect size, and the combined effect size is −0.51 (Z = 5.84, *P* < .01), statistically significant. That is, intravenous injection of parecoxib sodium for pain relief after knee or hip joint surgery can reduce the 24-hour resting VAS score compared with intravenous placebo (Fig. 7).

#### 3.3.7. 48-hour resting VAS score

**Figure 8.**
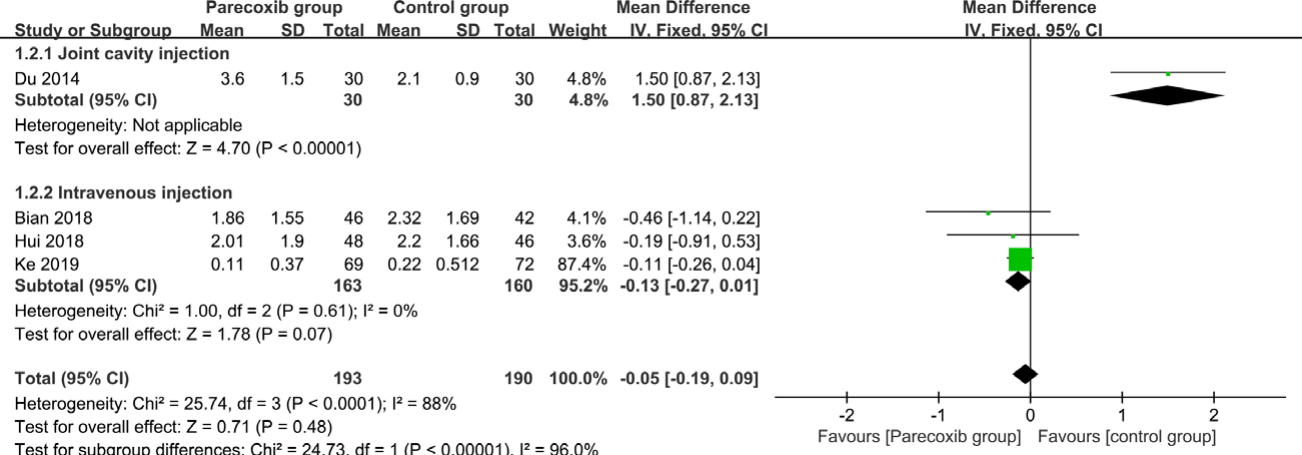
Forest plot of 48 hours resting VAS score. VAS = Visual analogue scale.

The control group was divided into 2 groups according to different administration methods, 1 group was intraarticular injection, and the other group was intravenous injection. The heterogeneity of the intravenous injection group (*I^2^* = 0%, *P* = .61 > .1) is not statistically significant, and the fixed effect model is selected to combine the effect size, and the combined effect size is −0.05 (Z = 1.78, *P* = .07 > .05), the 48-hour resting VAS score was not statistically significant. That is, compared with intravenous injection of placebo, intravenous injection of parecoxib sodium for pain relief after knee or hip joint surgery can reduce the 48-hour resting VAS score, but the reduction does not reach statistical significance. That is, from a statistical point of view, there is no difference between the 2 (Fig. 8).

## 4. Discussion

Pain is a complicated physiological and psychological activity, which includes the pain sensation caused by nociceptive stimuli on the body and the pain response of the body to nociceptive stimuli.^[34]^ Incision pain is a common acute pain caused by nociceptive, ischemic, and inflammatory mechanisms as well as nerve injury. Both TKA and THA can cause incision pain after operation, and the management of postoperative pain has a direct bearing on the physiology and psychology of patients.^[35,36]^ The results of the baseline population suggested that the objects of total knee or THA are elderly patients, the body and physiology of aging patients are in a declining stage, and the recovery time is slower than that of adults. Therefore, how to achieve effective management of preoperative pain and let patients put into the rehabilitation plan as soon as possible is more important.^[37]^

After operation, except that the injured cells released inflammatory mediators such as histamine and bradykinin, immune cells were attracted to the injured site and released proinflammatory cytokines (TNF-a, IL-1b, IL-6), which increased the expression of inducible cyclooxygenase (COX)-2 in monocytes, macrophages, fibroblasts, chondrocytes, and endothelial cells from 10-fold to 80-fold.^[36]^ Pareoxib sodium belongs to cyclooxygenase (COX)-2 inhibitor, which plays a reverse regulatory role. The most frequent adverse events after operation are nausea and vomiting. Severe nausea and vomiting may not only affect the comfort of patients but also lead to a variety of other complications, such as surgical suture cracking, bleeding, and so on.^[38]^ Nausea and vomiting not only reduces patients’ satisfaction with the health care system, but also prolongs hospital stay and health care costs.^[39]^ The safety of pareoxib sodium in postoperative pain relief of total knee joint and total hip joint is relatively stable. As can be seen from figure II, although the overall incidence of adverse events and the incidence of nausea and vomiting events were not statistically significant, they may also be superior to the placebo group to some extent. There was mild heterogeneity in overall adverse events and nausea and vomiting events, considering differences in adverse event statistics and placebo use in different studies. Pareoxib sodium belongs to COX-2 inhibitors. COX-2 inhibitors play an analgesic role by reducing the synthesis of peripheral prostaglandins to reduce inflammation and inhibit the expression of peripheral and central COX-2, and ultimately reduce the sensitivity of the central nervous system.^[40]^ A meta-analysis shows that perioperative use of pareoxib sodium can reduce pain and opioid intake in patients with TKA, and there are no serious complications,^[41]^ which is consistent with our study.

Due to the different opinion doses given during initial anesthesia and the combination of other painkillers, there is a 100% heterogeneity in the quantitative analysis of cumulative opioid consumption, so the researchers did not make a meta-analysis of opioid intake. However, from the related studies, it can be seen that the combined use of pareoxib sodium for pain relief can significantly reduce the consumption of opioids.^[20–23,28,32]^ Orthopedic surgeons account for a large proportion of the prescription of opioids for the management of postoperative pain. How to minimize the use of opioids and effectively control postoperative pain is a long-term topic. Although dozens of evidences supporting nonopioid analgesia, there is still no multimodal scheme that can completely eliminate the use of opioids. In this study, the combined use of pareoxib sodium reduced the use of opioids to some extent, which is of great clinical significance.

Since total knee or hip surgery usually improves a patient’s mobility, it is important in order to assess postoperative pain. The purpose of this study was to determine whether the use of nonopioid analgesia regimens can effectively control VAS pain score after total knee or hip surgery. Due to the inconsistency of exercise intensity, there is a great heterogeneity in the results of dynamic 24-hour and 48-hour VAS, and it is difficult to get a unified conclusion, so the researchers do not make too many comments on this content.^[20,24,25,31]^ The results of another study were about 24-hour and 48-hour resting VAS score, although only 4 articles were eventually included in the study, but there was no significant difference in the baseline VAS of the 4 studies. One of the 4 studies used articular pain relief as a placebo and the other 3 placebos were injected intravenously, so a subgroup analysis was performed. After subgroup analysis, it was found that pareoxib sodium showed the greatest advantage in the role of 24-hour resting VAS score, which could significantly improve the pain of patients. Another 48-hour resting VAS score was not statistically significant, but the outcome showed that pareoxib sodium was still beneficial in relieving pain. The analgesic effect of pareoxib sodium is similar to that of placebo after 2 days, so there is no need to further extend the analgesic time of pareoxib sodium. The description of pareoxib sodium manual describes limited clinical experience after 3 days of use, and the results of this study partly suggest that it is not necessary to use pareoxib sodium for >3 days.

The major limitation in this study is that the small sample size weakens our analysis results. The inconsistency of adverse events included in each study is an important reason for the heterogeneity of overall adverse events associated with pareoxib sodium. The time of the study is short and lack of long-term clinical significance. The placebo for postoperative pain relief of the knee joint or hip joint is mostly given intravenously, and more research is needed to support the analgesic effect of intracranial injection.

## 5. Conclusion

Our result suggest that he combination of pareoxib for pain relief did not lead to an increase in adverse events and the analgesic effect of combined use of pareoxib sodium was the most obvious 24 hours after operation. In order to further confirm our conclusions, more high-quality studies need to be carried out to verify them.

## Acknowledgments

We thank Jinlong Xu, Caimu Wang, Hai-Yan Xie, who have been a source of encouragement and inspiration.

## Author contributions

Each author has undertaken all of the following tasks listed:

conceived or designed the study, or both;

drafted the review or commented on it critically for intellectual content;

provided final approval of the document to be published.
